# Conceptualisation of a novel cognitive process in obsessive-compulsive disorder: A Delphi study on excessive rationalisation

**DOI:** 10.12688/wellcomeopenres.26104.2

**Published:** 2026-07-04

**Authors:** Elizabeth J. Kirkham

**Affiliations:** 1 Clinical and Applied Psychology, The University of Edinburgh School of Health in Social Science, Edinburgh, Scotland, UK

**Keywords:** obsessive-compulsive disorder; Delphi; thought control; participatory research; co-production; lived experience

## Abstract

**Background:**

Obsessive-compulsive disorder (OCD) is a common mental illness which involves intrusive thoughts and compulsive behaviours. Thought control strategies are a common factor in OCD, yet they are understudied and under-addressed in existing treatments. Excessive rationalisation is a novel conceptualisation of OCD-related thought control, describing a stressful process of arguing with or attempting to explain away unwanted thoughts. Investigation of this concept could offer a new perspective on the cognitive experience of OCD which may be missed or unappreciated within existing treatments.

**Method:**

The Delphi method was used to develop a pool of items that could describe this new concept. Twenty people with lived experience of OCD took part in four rounds. In the first round, responses to open-ended questions were used to generate a list of items which described the concept. In Rounds 2 and 3 participants rated the items so as to refine the initial list. In Round 4 participants ranked the items in order of how well they described excessive rationalisation.

**Results:**

Across the rounds a total of 61 items were generated. Thirty-one items were then discarded, either because they did not reach consensus, did not describe excessive rationalisation, or were redundant. Ninety per cent of participants (strongly) agreed that the final pool of 30 items described the concept of excessive rationalisation.

**Conclusions:**

The present research drew on lived experience to conceptualise a novel concept which may be important for understanding OCD and its persistence.

## Plain Language summary

Obsessive-compulsive disorder (OCD) is a common mental illness that causes substantial distress to those who live with it. People with OCD typically experience frequent unwanted thoughts (called “obsessions”) and feel that they have to perform specific behaviours (called “compulsions”). While treatments for OCD can be very effective for some people, a large minority of people do not benefit from existing treatments. It is therefore important to understand more about OCD and how it works.

The present research drew on previous findings that people with OCD try harder than people without OCD to control their unwanted thoughts. Conversations with people with OCD revealed that trying to argue with or disprove unwanted thoughts is a common strategy for those with the condition. We termed this strategy “excessive rationalisation”.

The present research was designed to create a list of statements that describe excessive rationalisation. It used the Delphi method, which is a research method that is used to generate new knowledge by combining the expertise of a group of people. Specifically, 20 people with personal experience of OCD were invited to share their views on excessive rationalisation using an online questionnaire. The researcher then drew on their responses to generate a draft list of statements. After this, participants were asked to rate the statements according to how well they represented the concept of excessive rationalisation. Over three online surveys the list of statements was refined, leaving a final list of 30 statements. This list of statements can be used in future work to create a questionnaire that measures excessive rationalisation. Measuring excessive rationalisation could help researchers to learn more about OCD and why some people do not benefit from existing treatments.

## Introduction

Obsessive-compulsive disorder (OCD) is a common mental health condition which affects approximately 2% of people and is associated with substantial illness burden globally ( Kalanthroff & Wheaton, 2022). People with OCD typically experience intrusive irrational thoughts known as obsessions, and engage in time-consuming behaviours known as compulsions. The most well-established treatments for OCD are cognitive behaviour therapy (CBT) and selective serotonin reuptake inhibitors ( Juel et al., 2025).

While there is extensive evidence in support of CBT over placebo ( Öst et al., 2015), a substantial portion of people with OCD do not respond successfully to existing treatments ( Atmaca, 2016). A meta-analysis by Öst et al. (2015), found that less than half of participants (43.4%) who received CBT for OCD experienced a clinically significant improvement in symptoms. Similarly, in a real-world study of 213 people seeking treatment for OCD, Eisen et al. (2013) found that after five years only 12% of people were in remission with a further 5% in partial remission. Research is therefore required to understand why a substantial portion of people with OCD do not respond adequately to existing treatments.

The role of cognition in OCD has long been recognised, with seminal work by the Obsessive Compulsive Cognitions Working Group (OCCWG) highlighting the role of dysfunctional beliefs in maintaining the condition ( OCCWG, 1997; OCCWG, 2001). Maladaptive beliefs among people with OCD include inflated responsibility, overestimation of threat, and excessive concern about the need to control one’s thoughts. Various psychological instruments have been developed to assess such beliefs, including the Obsessional Beliefs Questionnaire and the Interpretation of Intrusions Inventory (OCCWG, 2001). The maladaptive belief that they must control their thoughts appears to influence the cognitive strategies people with OCD use ( Purdon et al., 2007). Therefore it is not just the belief that they must control thoughts that maintains the condition, but the mechanism by which they put that belief into practice. Studies have found that maladaptive attempts to control or suppress unwanted thoughts are related to increased severity of OCD and reduced success of treatment ( Abramowitz et al., 2003; Allen et al., 2016; Harsányi et al., 2014; Lee et al., 2016; Purdon et al., 2007).

Despite the likely importance of thought control strategies in OCD, commonly used measures of OCD rarely address such concepts ( Purdon et al., 2007). For example, thought control strategies are not covered by the Obsessive-Compulsive Inventory ( Foa et al., 1998) or the Maudsley Obsessional Compulsive Inventory ( Hodgson & Rachman, 1977). While the Yale-Brown Obsessive Compulsive Scale ( Goodman et al., 1989) does address effortful resistance to OCD symptoms, it does not explicitly address attempts to control the thoughts via engagement with them ( Purdon et al., 2007). A similar pattern can be found in many existing approaches to OCD treatment, with some treatment manuals focusing more on overt compulsions (such as washing) over the cognitive elements of the condition (such as self-reassurance) ( Lee et al., 2016). Therefore, a closer focus on maladaptive thought control strategies in OCD could enhance our understanding of the condition and potentially contribute to more effective treatment strategies.

Two questionnaires which have been used to measure maladaptive thought control in general are the Thought Control Questionnaire ( Wells & Davies, 1994) and the White Bear Suppression Inventory (WBSI; Wegner & Zanakos, 1994). The Thought Control Questionnaire measures five strategies for controlling thoughts: punishment, worry, reappraisal, social control and distraction. Punishment and worry are considered maladaptive strategies, and have been positively associated with OCD symptoms ( Abramowitz et al., 2003; Lee et al., 2016). The WBSI is designed to measure attempts to suppress unwanted thoughts ( Nabors et al., 2022). Though the WBSI is not specific to OCD, there is some evidence that more OCD obsessions are associated with higher scores on the WBSI ( Harsányi et al., 2014).

This research suggests that thought control strategies may be a valuable route for understanding OCD and its maintenance. However, as the aforementioned measures of thought control are focused on psychopathology more generally rather than OCD specifically ( Wells & Davies, 1994; Nabors et al., 2022) there is scope for further investigation within this field. In light of this, and drawing on her own lived experience, the researcher sought to develop a concept which could address the gap in current conceptualisations of thought control in OCD. This new concept was termed “excessive rationalisation”. Excessive rationalisation refers to a mental strategy used by people with OCD in which they try to rationalise away the fear provoked by an intrusive OCD thought. It could also be conceptualised as arguing with an OCD thought. For example, an unwanted OCD thought might be, “I left a lamp plugged in when I went out, it could catch fire and kill my pets.” An excessive rationalisation response might involve coming up with reasons why the feared response wouldn't happen, such as “it is a new lamp that has been tested to high safety standards”, or “my pets are in a different room and even if the lamp caught fire the firefighters would have time to save my pets.” On the surface this appears similar to the therapeutic technique of cognitive reappraisal, in which individuals attempt to re-evaluate how they think about distressing situations ( Clark, 2022). However, in the case of excessive rationalisation, attempted reappraisal does not meaningfully reduce distress ( Ford & Troy, 2019; Johnstone et al., 2007). The aim of the present research was to begin operationalising the concept of excessive rationalisation by drawing on the lived experience of those who encounter it.

## Method

### Procedure

Before starting this project, the researcher sought expert input from people with lived experience of OCD through two online stakeholder sessions. The purpose of these sessions was to verify that the concept of excessive rationalisation was meaningful, and to refine its definition (for example, there was discussion on whether excessive rationalisation is automatic or effortful). People who attended the meetings were paid community stakeholders rather than participants, and as such no data were collected. Rather, the discussions were used to inform initial development of the new concept. The researcher used this discussion to inform the way excessive rationalisation was described going forward.

Once an initial concept had been delineated, a Delphi study was used to generate a pool of items which described it. The Delphi method is a research method in which a group of people anonymously contribute their ideas across multiple “rounds”, with the purpose of reaching consensus on a new piece of knowledge ( Beiderbeck et al., 2021). This method was chosen firstly because it allows for the systematic distillation of knowledge on a new or under-studied topic ( De Villiers et al., 2005), and secondly because it provides a platform for co-producing knowledge with people who have lived experience of the topic being studied ( Kirkham et al., 2021). This process therefore represented a unique opportunity to capitalise on “insider” knowledge while maintaining a scientifically rigorous approach. The following sections describe this Delphi process.

The research project received a favourable opinion from the University of Edinburgh School of Health in Social Science Ethics Committee (REF: CAHSS2307/02). Recruitment involved contacting individuals who had previously expressed an interest in OCD research, and advertising on social media. Individuals were invited to participate in the Delphi study if they had lived experience of OCD and felt that the concept of excessive rationalisation was relevant to their own experiences. They were also required to be over 18 and available to complete four surveys during the study period. The people who took part in the Delphi were different to the community collaborators who took part in the earlier conceptual discussions. Surveys for each round of the Delphi were sent by email. Each survey was open for one week. Participants were sent up to two reminder emails during this time. At the beginning of each survey participants were presented with a participant information sheet. Participants provided written consent before every round of the Delphi by agreeing to a series of “tick box” consent statements. Participants were offered £15 for each completed survey, with most completing all four surveys and being eligible for the full £60.

### Screening

As the purpose of this research was to co-design items that could measure excessive rationalisation, a screening form was used to identify participants who felt they had experience of excessive rationalisation. Individuals who opened the screening form were given the opportunity to read the Participant Information Sheet, and were provided with a description of excessive rationalisation (Appendix 1). After reading this description, potential participants were asked if they felt that excessive rationalisation could be relevant to their own experience, and if they had personal experience of OCD. Those who stated that it was not relevant to their experience, or that they did not have experience of OCD, were thanked for their time and screened out of the survey. Remaining participants were asked to provide their email address. Unfortunately it is not possible to accurately report the number of interested individuals who were screened out, as the majority of responses to the screening form appeared to be from bots – while the pattern of genuine participants signing up was relatively steady and sporadic, on one day there were batches of suspected bot responses at specific times (e.g. 19 responses at 12:21, 20 responses at 12:26 etc.).

Regarding sample size, Delphi studies are required to balance the benefits of a larger sample with the ability to implement rapid and thorough analysis, especially in relation to qualitative data ( Hasson et al., 2000). Jorm (2015) suggest that 20 is an appropriate sample size for reaching consensus within a Delphi study. To this end the present study aimed to recruit between 20 and 35 participants, bearing in mind the potential for attrition between surveys.

### Round 1

All registered participants (n = 27) were sent a link to the first survey at the same time. Participants were asked to complete brief demographic information (gender, age, ethnicity and country), and to state whether they had any mental health conditions other than OCD. They then indicated whether their OCD had been diagnosed by a professional or whether it was self-diagnosed. Following this, they were asked questions about their experiences of treatment for OCD. When answering these questions participants were asked to consider any therapeutic approach that was intended to treat their OCD, whether or not that approach had been used to treat another condition as well (i.e. medication for OCD and depression). Participants were asked to indicate which of the following categories of treatment they had received for their OCD: CBT, medication, or another form of therapy. Those who had received CBT were asked to estimate how many courses of CBT they had received. Participants were advised that a course of CBT referred to multiple sessions, and that they should enter the number of full courses, not the number of individual sessions. Participants who had received medication for their OCD were asked to estimate how many different medications they had tried. Participants were also asked to complete the Obsessive-Compulsive Inventory Revised (OCI-R) ( Foa et al., 2002).

Participants were then provided with a description of excessive rationalisation (Appendix 2). After reading the description, participants were asked if excessive rationalisation was something they had experienced or engaged in, whether recently or in the past. The response options were “Yes, definitely”, “Yes, I think so”, “I don’t know”, or “No, I have never experienced it.” The survey was set up so that individuals who responded “No, I have never experienced it” were screened out of the rest of the survey (£15 compensation for taking part in this survey was still offered). Similarly, the survey was set up so that participants who responded “I don’t know” were informed that the remainder of the survey would ask about excessive rationalisation, and were given an option to leave the survey if they wished (whilst still receiving £15 compensation).

The next section of the survey included a range of open-ended questions designed to elicit participants’ perspectives on what excessive rationalisation might involve. Briefly, these questions covered topics such as what thoughts and feelings they experience when engaging in excessive rationalisation, how excessive rationalisation differs from other concepts such as rumination, worry, and cognitive reappraisal, how excessive rationalisation relates to OCD, and what key elements should be measured. Participants were also asked to indicate whether excessive rationalisation is best described as “something people engage in”, “something people experience”, “either of the above”, or “something else”. Participants who chose “something else” were invited to specify what this should be.

Once the data had been collected a rapid thematic analysis of the text responses was carried out. Each response to each question was read, and codes were generated to describe each key point within a participant’s answer. These codes were then grouped into themes. For example, the codes “debating”, “apply logic to intrusive thoughts”, and “aim to disprove thoughts” were classified under the theme “using logic”. These themes were then used to generate an initial list of statements which described excessive rationalisation (Appendix 3).

### Round 2

Participants were presented with a definition of excessive rationalisation to remind them of the concept. Following this, they were presented with the items ( *n* = 58) generated by the analysis in Round 1. Participants were informed that the items were draft statements that could be used in a new questionnaire designed to measure excessive rationalisation. The items were presented in six blocks, the order of which was counterbalanced across participants. Participants were asked to rate how well each item described excessive rationalisation using a 6-point scale which ranged from “Not well at all” (1) to “Very well” (6). A space for optional comments was provided after each item. Four items were designed to be reverse-coded, should they be included in a questionnaire measure of excessive rationalisation. Each of these items was followed by the statement “Please note that if this statement is included in the questionnaire measure of excessive rationalisation it will be reverse coded (i.e., if the participant agrees with the statement then it will indicate a low level of excessive rationalisation).” After rating all the items, participants were asked whether any element of excessive rationalisation was missing from the existing list of items.

Analysis of the data sought to identify which statements should be included in the item pool, which should be excluded, and which should be re-rated in Survey 3. Consensus for retaining an item was set as a median rating (across participants) of > = 5 and an inter-quartile range < = 1.5. Inter-quartile range was used based on best practice recommendations for assessing consensus in Delphi studies ( Beiderbeck et al., 2021). Consensus for discarding an item was set as a median of <= 3 across participants and an inter-quartile range < = 1.5. Items which did not reach consensus were retained and presented again in Survey 3.

### Round 3

**Re-rating items which did not reach consensus in Round 2.** In Survey 3, each item which had not reached consensus in Round 2 was presented again, along with a sentence informing participants of the median response in the previous round (e.g. “The average response in the last survey was: “Very well”). Participants were asked to rate the item again. Items which reached consensus (median > = 5, IQR > = 1.5) were retained.

**Removal of redundant items.** The research aim was to create a succinct list of items which could measure excessive rationalisation. However, during analysis of Survey 2 it became apparent that a large number of items were likely to be retained. Therefore, in Survey 3, items which had reached consensus in Survey 2 were presented as pairs, and participants were asked to rate the similarity of the two statements in each pair. Participants selected one of five statements which ranged from “They have completely different meanings” to “They have essentially the same meaning”. Each participant was presented with 15 pairs. This allowed for three or four individual ratings for each of the 78 possible pairs while minimising the burden on participant time.

For analysis purposes a pair of items was considered “very similar” if it had a median similarity rating > = 4. If an item was rated as “very similar” to at least two other items it was flagged for redundancy investigation, which involved the following procedure. Specifically, where an item was listed as very similar to another item, the two mean ratings from Round 2 were compared, and the item with the higher mean was noted. An item was discarded if it had the lower mean in every pairing it appeared in.

### Round 4

One goal of Round 4 was to identify redundant items. By Round 4 there were 34 items, resulting in 473 possible pairs of items (in addition to the 78 pairs already rated in Round 3). As it would not be possible to ask participants to rate this many pairings, other options were explored. Generative AI was considered, but its ratings were not reliable. Therefore, a researcher gave each of the remaining 473 pairs a rating of 1 to 5, with 1 representing “They have completely different meanings”, and 5 representing “They have essentially the same meaning.” These ratings are accessible as part of the dataset associated with this study. Pairs which were rated as either 4 or 5 (n = 76) by the researcher were entered into Survey 4 for rating by the participants. Each participant was asked to rate one block of pairs, with each block containing either 15 or 16 pairs.

Redundancy was investigated using the same procedure as Round 3 with the following additional considerations. If a statement had reached consensus in Round 2, its Round 2 mean was used. If a statement had reached consensus in Round 3, its Round 3 mean was used. In cases where both items from a “very similar” pair in Round 3 had been carried forward (i.e. neither was discarded), this pair was included in the Round 4 analysis. This was due to the fact that pairs from Round 3 were not re-assessed in Round 4, so bringing them forward allowed similar items that were in the Round 3 set to be assessed alongside items that were in the Round 4 set.

In addition to identifying redundant items, participants were asked to rank the list of statements in order of how well they represented excessive rationalisation. The starting order of statements was randomised across individuals. Participants were also asked to indicate which response scales would be most appropriate for an eventual excessive rationalisation questionnaire (for example, “not at all” to “extremely”; “strongly disagree” to “strongly agree”), and what time frame respondents should consider when completing this questionnaire (for example, past month, past two weeks). Finally they were asked to indicate how well the final list of items represented excessive rationalisation as a concept.

## Results

### Participants

**Figure 1.**
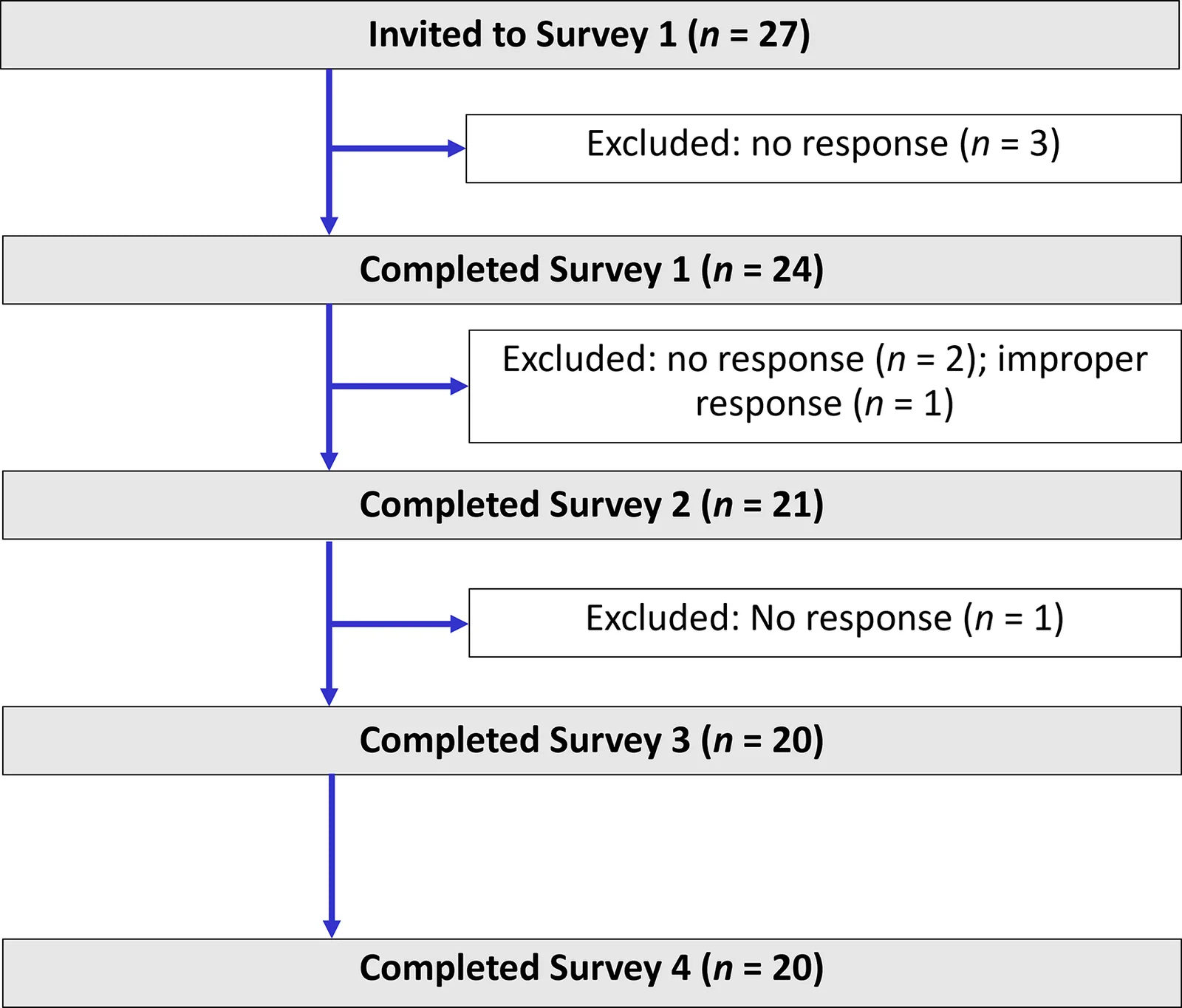
Flow diagram of responses to the surveys. *Note*: One response to Survey 1 was considered “improper” because all the qualitative questions (comprising the majority of the survey) were left blank.

Figure 1 illustrates response rates across the four surveys. One participant completed the first survey twice. It was decided that all the qualitative data provided by this participant (across the two Round 1 surveys) would be included, but that only their first response would be used for the quantitative data. One participant completed the first survey but left all the qualitative questions blank. As the qualitative questions were the core of the survey, this participant was excluded due to suboptimal focus on the task.

Demographic data for the 23 participants who took part in at least one survey are presented below. Nineteen participants were female and four were male. Participants were aged between 24 and 55. The mean age was 33.70 ( *SD* = 8.15). Participants self-described their ethnicity. Ethnicity was then categorised according to UK Census categories to protect participants’ identities. Following these Census categories, 17 participants were White, three had Mixed or multiple ethnicities, two were Asian, and one was within the “Other” category. Nineteen participants lived in the United Kingdom, two lived in the USA, one lived in Indonesia and the final participant lived in Portugal.

A majority of participants ( *n* = 17, 74%) had other mental health conditions in addition to OCD. This included anxiety ( *n* = 14), depression ( *n* = 12), PTSD ( *n* = 2), eating disorder ( *n* = 2), Tourette’s syndrome ( *n* = 2), borderline personality disorder ( *n* = 1), and bipolar disorder ( *n* = 1). The survey did not explicitly ask for information on neurodivergence, however a number of participants chose to mention this when provided with an open text box asking about comorbid mental health conditions. Three participants stated that they were autistic, and an additional three participants stated that they had ADHD.

Of the 23 participants, 21 stated that they had been diagnosed with OCD by a professional, and the remaining two had self-diagnosed OCD. The mean number of years that participants had lived with OCD was 18.70 ( *SD* = 11.00). The mean OCI-R score was 32.57 ( *SD* = 11.69).

Participants were asked to indicate which types of OCD treatment they had ever received. Most participants ( *n* = 18; 78%) had received CBT. Fifteen participants (65%) had received medication, and 12 participants (52%) had received another form of therapy. Nine participants (39%) had tried all three of these types of treatment (CBT, medication, and other therapy) at some point in their lives. Participants who had received CBT were asked to indicate how many courses of CBT they had received (i.e. number of courses of treatment, not number of individual sessions). The median number of courses of CBT was three (range = 1–15 courses). Participants who had received medication for OCD were asked to indicate how many different medications they had tried. The median number of medications tried was two (range = 1–8 medications).

### Round 1

When asked whether excessive rationalisation was something they had experienced, 17 participants responded “Yes, definitely”, and the remaining six participants responded “Yes, I think so”. Regarding the way in which excessive rationalisation should be discussed, seven participants (32%) felt it was something people experience, seven (32%) felt it was something people engage in, and eight (36%) felt either description was appropriate. It was therefore decided that both forms of terminology were relevant to excessive rationalisation.

Rapid thematic analysis was used to generate codes from the qualitative data. These codes were categorised into themes. The themes were then used to generate a list of 58 items (Appendix 3) for use in Round 2. A large number of items was preferred as the purpose of this round was to capture as many facets of the excessive rationalisation concept as possible.

### Round 2

Of the 58 items, 13 items reached consensus for inclusion in the first list of items describing excessive rationalisation (Appendix 3). Six items were discarded based on analysis of qualitative comments. Of these, three were discarded because they described OCD itself (not excessive rationalisation), one was discarded because it described cognitive reappraisal, another because it was too vague, and a final item was discarded because its wording included a double negative. Thirty-five items did not reach consensus and were therefore retained for re-rating in Survey 3. Two of these 35 items received minor edits for clarity (Appendix 3).

Although participants were informed when an item was designed to be reverse-coded, it was apparent from participant responses that the instructions for rating reverse-coded items had been unclear. Therefore, it was decided that the four reverse-coded items would be presented again in Survey 3 with additional explanatory text. The explanatory text was designed to make it clearer that the statements were purposefully designed to describe the opposite of excessive rationalisation (i.e. they would be reverse coded if included in a questionnaire), and that an item which clearly described the opposite of excessive rationalisation should be rated highly.

Based on analysis of comments at the end of the survey, three new items were generated. These were: “Arguing with my unwanted thoughts is something that happens automatically”, “I automatically argue with my unwanted thoughts”, and “Arguing with unwanted thoughts is something I do automatically”.

### Round 3

**Re-rating items which did not reach consensus in Round 2.** During Round 3, participants rated a total of 42 statements. This included the 35 items which had not reached consensus in Round 2, the three items added following qualitative analysis in Round 2, and the four reverse-coded items. Of these 42 statements, 23 reached consensus for inclusion and were retained for Round 4.

**Removal of redundant items.** The 13 items that had been selected for inclusion during Round 2 were paired together to create 78 pairs. Nine of these pairs received median similarity ratings > = 4 (“very similar”). Examination of these pairs revealed six individual items which were rated as “very similar” to at least two other items. Of these items, two were classified as redundant (Appendix 3) following the procedure detailed in the Method section.

### Round 4

**Removal of redundant items.** Redundancy of items was investigated using the procedure described in the Methods. This resulted in four items being discarded due to high levels of similarity with other items.

**Supporting features of a draft questionnaire.** Participants were asked to select which rating scales would fit well with the excessive rationalisation items ( Table 1). Multiple responses were allowed. Scale options B and E were equally highly rated by participants. Participants were also asked to consider the introductory text for an eventual excessive rationalisation questionnaire. A majority of participants felt that people completing an excessive rationalisation questionnaire should be asked to consider their thoughts/feelings in general ( *n* = 11) rather than in the past month ( *n* = 6) or the past two weeks ( *n* = 2).

**Table 1.** Participant choice of response scale for the draft excessive rationalisation questionnaire.

Option	Response scale	Participant votes
A	Not at all/ A little/Moderately/A lot/Extremely	7
B	Never/Rarely/Sometimes/Very often/Always	9
C	Not at all/Several days/More than half the days/Nearly every day	3
D	Strongly disagree/ Moderately disagree/ Slightly disagree/ Slightly agree/Moderately agree/Strongly agree	6
E	Very untrue of me/Untrue of me/ Somewhat untrue of me/Neutral/Somewhat true of me/True of me/Very true of me	9

**Suitability of the draft questionnaire.** Participants were presented with a brief description of excessive rationalisation as a concept (Appendix 1). When asked whether they agreed that the draft questionnaire measured the concept of excessive rationalisation eight responded “strongly agree”, 10 responded “somewhat agree”, one responded “neutral” and one responded “strongly disagree”.

**Ranking of items.** At the beginning of Round 4 there were 34 items marked for inclusion in the draft questionnaire (four items were removed during analysis of Round 4 responses, so the final item pool contained 30 items). Participants were asked to rank the items in order of how well they measured excessive rationalisation, with 1 being the best and 33 being the worst ( Table 2). One item was reverse-coded and was not included in the list of items to rank in order to avoid confusion.

**Table 2.** Ranking of items included in Round 4.

Rank	Item	Mean (SD) ranking	Median ranking
1	I spend a lot of time coming up with reasons why my fears are unlikely to happen	12.21 (8.05)	11
2	I argue with my unwanted thoughts because I want them to go away	12.89 (9.13)	11
3	I get into stressful debates with my own thoughts	13.47 (8.06)	11
4	I find myself trying to get rid of unwanted thoughts by arguing with them	14.11 (8.65)	14
5	I try to cope with unwanted thoughts by finding reasons why they are not true	15.00 (9.14)	12
6	When I have unwanted thoughts I try to reason with them	15.00 (8.94)	13
7	Coming up with rational responses to my worries rarely provides me with the relief I am looking for	15.68 (12.50)	11
8	Sometimes I cannot stop arguing with my own thoughts	15.74 (11.14)	12
9	When I try to disprove my unwanted thoughts I get stuck in a loop	16.26 (8.98)	17
10	I try to reduce feelings of uncertainty using logic	16.42 (8.95)	17
11	When I have a frightening thought I list reasons why it is not true	16.53 (8.59)	16
12	I try to find relief from my fears by reasoning with them	16.63 (11.41)	14
13	I use logic to try to reduce my feelings of distress	16.89 (8.05)	11
14	I get stuck arguing with unwanted thoughts in my mind	16.95 (9.65)	15
15	When I try to disprove my unwanted thoughts I find myself thinking about them even more	17.00 (11.70)	20
16	When I am afraid I use logic and reasoning to try to reassure myself	17.11 (8.59)	16
17	The more unwanted thoughts I have the harder I try to disprove what they say	17.21 (9.17)	18
18	I try to reason with unwanted thoughts to stop the fear taking over *	17.32 (8.63)	16
19	When I have unwanted thoughts I try to make myself feel better by arguing with them	17.42 (8.62)	19
20	When I am arguing with my thoughts it feels endless	17.47 (10.32)	17
21	I continue arguing with my thoughts even when it is not helping me feel better	17.47 (10.65)	19
22	When I have unwanted thoughts I try to solve them	17.79 (10.10)	20
23	I try to disprove my worries using facts or statistics	17.84 (8.95)	19
24	I have arguments with myself in my mind *	17.89 (11.82)	19
25	I argue with my unwanted thoughts *	18.26 (9.39)	20
26	When I try to disprove my fears I end up going back and forth in my mind *	18.37 (8.67)	16
27	I feel an urge to reason with my unwanted thoughts	18.68 (7.36)	18
28	Coming up with rational responses to my worries rarely provides me with the reassurance I want	18.68 (11.11)	22
29	Responding to my unwanted thoughts is exhausting	18.89 (10.70)	18
30	I try to counteract my worries using logic	18.89 (9.52)	21
31	When I have unwanted thoughts I try to fix them	19.79 (8.07)	19
32	My logical replies to my worries never “stick” in my brain	19.95 (9.48)	21
33	I am more likely to argue with my unwanted thoughts when I am stressed than when I am calm	21.16 (8.62)	20
Not ranked	Arguing with my unwanted thoughts makes them go away **	NA	NA

*Note*:

* These items were removed after the ranking exercise due to similarity.

** This reverse-coded item was not included in the ranking exercise.

## Discussion

The present research used the Delphi method to co-produce a draft questionnaire measure of excessive rationalisation, a maladaptive cognitive strategy in which people with OCD attempt to argue with or explain away their unwanted thoughts. Twenty people with lived experience of OCD took part in four rounds of a Delphi study in order to generate a pool of items which describe this new concept. This study represents a foundational step in translating excessive rationalisation from a shared idea to a measurable construct.

Thought control strategies were a central component of the item pool with statements such as “When I have unwanted thoughts I try to reason with them”, and “I try to disprove my worries using facts or statistics”. This aligns with prior research identifying the importance of thought control strategies in OCD ( Lee et al., 2016; Abramowitz et al., 2003; Purdon et al., 2007). While prior questionnaire measures of thought control have focused on cognitive strategies across (anxious) individuals more generally ( Wells & Davies, 1994; Wegner & Zanakos, 1994), the present research provides an opportunity to observe this within OCD specifically. Items within the draft questionnaire highlighted the stressful nature of this cognitive strategy: “Responding to my unwanted thoughts is exhausting”, and “I get into stressful debates with my own thoughts”. This aligns with prior research which found that people who have difficulty suppressing thoughts experience increased negative affect when performing a thought suppression task ( Göbel & Niessen, 2021). It also fits with cognitive conceptualisations of OCD which emphasise that it is not the presence of intrusive thoughts themselves which is maladaptive, but the way in which the individual interprets and responds to them ( Myers et al., 2008; Rachman & de Silva, 1978).

It is pertinent to consider how excessive rationalisation may fit conceptually within wider understandings of OCD as a mental health condition. A core element of OCD is neutralisation, in which people use overt (e.g. hand washing) or covert/mental (e.g. repeating numbers, reciting prayers) strategies to help them manage the anxiety caused by their unwanted thoughts ( Belloch et al., 2015). Attempts at thought control or suppression have also been recognised within the framework of covert/mental neutralisation ( Belloch et al., 2015; Lee et al., 2016; Purdon et al., 2007). For example, Purdon et al. (2007) found that people with OCD used thought suppression as a form of neutralisation. In light of this it is possible that excessive rationalisation is best understood as a form of neutralisation. Of note, Belloch et al. (2015) found that patients who used more covert neutralisation strategies prior to CBT treatment experienced less improvement from CBT, highlighting the need for more research on the role of cognitive suppression in treatment response. Relatedly, high rates of prior experience with CBT among the present sample could have influenced participants’ perspectives on excessive rationalisation, as they have likely received prior training in recognising rationalisation-type responses. Future research could seek to examine how excessive rationalisation interacts with CBT and CBT outcomes. 

The importance of lived experience in mental health research is becoming increasingly recognised ( Fischer et al., 2024). To this end, it was crucial that initial attempts to measure a new mental health-related concept were grounded in the expertise of people who experience that phenomenon. Using the Delphi method allowed for this process to take place systematically ( Kirkham et al., 2021). While the centring of patient perspectives is a strength of this study, it is legitimate to question whether the inclusion of additional stakeholders, such as clinicians or researchers, could have strengthened development of this novel concept. Therefore further refinement of this new concept should seek to integrate a wide range of stakeholder perspectives. The present sample was also predominantly female, White, and UK-based. This may limit the cultural and demographic generalisability of the item pool.

Another potential limitation of the present study is that, while the initial discussions with collaborators involved (online) face-to-face discussion, the Delphi itself was conducted through online surveys. Including opportunities for discussion in the Delphi process could have helped confirm that the participants possessed a shared understanding of the intended concept. An unexpected issue that arose during the Delphi process was the emergence of a large number of items which were rated as conceptually relevant. To address this, measures were introduced in Rounds 3 and 4 to identify highly similar items (and thus limit item redundancy). Ideally, participants would have been asked to rate every pair. However, in order to minimise the time burden on participants a researcher completed a pre-assessment of similarity. It is possible that in doing so the researcher missed some similar pairs which would have been picked up if all pairs had been rated by participants.

Notwithstanding these limitations, this research has generated a pool of items pertaining to excessive rationalisation, a novel way of conceptualising thought control in people with OCD. The concept of excessive rationalisation builds upon existing work on maladaptive thought patterns in OCD, and highlights how prior conceptualisations of thought control can be adapted for understanding OCD. Identifying new directions for OCD research is of crucial importance, given the extent to which OCD continues to damage lives throughout the international community ( Kalanthroff & Wheaton, 2022). This work represents the first step in a broader programme of research. Future work will build on the present findings by undertaking formal scale development and clinical validation of the measure.

## Appendices

Appendices can be accessed in the “Supplementary material_WellcomeOpenResearch.docx” file at: https://doi.org/10.7488/ds/8118

Appendix 1: Brief Description of Excessive Rationalisation used in the Screening Form and Survey 4.

Appendix 2: Description of Excessive Rationalisation Provided in Survey 1.

Appendix 3: Table A1 Full list of items and outcomes.

## Data Availability

Kirkham, Elizabeth. (2026). Conceptualisation of a novel cognitive process in obsessive-compulsive disorder: A Delphi study on excessive rationalisation, [dataset]. University of Edinburgh. School of Health in Social Science. https://doi.org/10.7488/ds/8118. This project contains the following underlying data:
• Copies of surveys used in the project (including participant information sheets and consent forms): Screening, Survey 1, Survey 2, Survey 3, Survey 4 • De-identified data from the project • Supplementary materials: Appendix 1 Brief Description of Excessive Rationalisation used in the Screening Form and Survey 4; Appendix 2 Description of Excessive Rationalisation Provided in Survey 1; Appendix 3 Table A1 Full list of items and outcomes • Qualitative coding of Survey 1 (de-identified) • Researcher ratings of statements prior to Survey 4 • Draft excessive rationalisation questionnaire Data are available under the terms of the Creative Commons Attribution 4.0 International license (CC-BY 4.0). Preprint. The first preprint version of this article can be accessed at: https://osf.io/preprints/osf/79ca4_v1. This preprint was revised prior to submission to Wellcome Open Research.
