# Supplementary material for: Conceptualisation of a novel cognitive process in obsessive-compulsive disorder: A Delphi study on excessive rationalisation
Source: Wellcome Open Res. 2026 Jul 4;11:282. Originally published 2026 May 13. [Version 2] doi: 10.12688/wellcomeopenres.26104.2 (PMC13555087; doi:10.12688/wellcomeopenres.26104.2)
Supplement: Peer review history

# Reviewer report: Reviewer response for version 2

**DOI:** 10.21956/wellcomeopenres.29897.r159872

**Published:** 2026-07-17

**Authors:** Marketa Ciharova

**Affiliations:** 1 Vrije Universiteit Amsterdam, Amsterdam, North Holland, The Netherlands

I am happy with the current revision.

Is the work clearly and accurately presented and does it cite the current literature?

Partly

If applicable, is the statistical analysis and its interpretation appropriate?

Not applicable

Are all the source data underlying the results available to ensure full reproducibility?

Yes

Is the study design appropriate and is the work technically sound?

Yes

Are the conclusions drawn adequately supported by the results?

Yes

Are sufficient details of methods and analysis provided to allow replication by others?

Partly

Reviewer Expertise:

Psychotherapy, randomized controlled trials, meta-analysis, common mental disorders

I confirm that I have read this submission and believe that I have an appropriate level of expertise to confirm that it is of an acceptable scientific standard.

# Reviewer report: Reviewer response for version 1

**DOI:** 10.21956/wellcomeopenres.28746.r157956

**Published:** 2026-06-25

**Authors:** Marketa Ciharova

**Affiliations:** 1 Vrije Universiteit Amsterdam, Amsterdam, North Holland, The Netherlands

Dear author,

 Thank you very much for the invitation to review your important paper. In the current study, you conducted a Delphi-method study, where in 4 rounds, you created a list of items serving as a based for a future questionnaire assessing excessive rationalisation in obsessive-compulsive disorder (OCD). As such, the current study is definitely a start of new exploration of symptoms of OCD, which may, in the longer term help improvement of interventions for this disorder. That being said, I have several points that I hope could help you improve the manuscript even further, so that the reader gains even more knowledge from your study:

 Dear editor,

 Thank you very much for your invitation to review this paper. I hope my comments with contribute to the improvement of the current manuscript.

 Abstract:

 Major comments: It would be helpful to explain in one or two sentences what the background of research on excessive rationalization is and why it is important to create and study this concept, instead of focusing on, for example, optimization of existing treatments for OCD.

 Plain Language Summary:

 Minor comments: “It is therefore important to understand more about OCD and how it works.” => Could you perhaps reformulate this sentence? I feel like “how it works” is a bit too general and informal for a peer-reviewed journal.

 Introduction:

 Major comments:

- Page 4, second last paragraph: You are referring to having held two meetings with paid community collaborators in an effort to develop the concept of excessive rationalisation, which eventually also coined this term. Were these meetings held by your research group, or by other researchers? If the latter, I would recommend adding a reference to this event (a study resulting from this event, or a personal communication). If it was held by your research group, I would say that it was already the first step of the current research, so it would be best of describe this in the Methods, and its Results in the Results, if it fits well. Alternatively, if there is already a study published, then you can use a reference to this study here.
- Pages 4-5: I would also place the whole explanation of what Delphi study is and why to conduct it in the Methods. I would just jump in the Introduction from the background of the research question to the aims of the current study, and make sure that all methods are described in the Methods.
- Page 5: Similarly, I would completely remove the last paragraph of the Introduction and explain it in the Methods. The Introduction should be closed by a paragraph summarising the aims of the current study.
- I also think that the Introduction does not really clearly defined excessive rationalisation, so the reader gets a bit lost what the manuscript is actually about. Maybe you could place some of the explanations from Appendices 1 and 2 to the Introduction, because they illustrate very well what the manuscript focuses on.

Methods:

 Major comments:

- Screening: Were there other in/exclusion criteria for the participants, such as age?
- Round 1: I feel like the pre-survey period is very extensively described, even though it is not really the focus of the current paper. Maybe you can only say that an inclusion criterion of the current study is that participants reported experiencing excessive rationalisation, and then move on to describe the participation of the study by the participants who met this criterion?

Results:

 Major comments:

- The paper states that 27 participants registered their interest, but then in the Results, it is written that 23 individuals participated. What happened with the 4?

Discussion:

 Major comments:

 • ⁃ In the summary of the results, you are mentioning three themes that the included items can be categorised to: thought control, attempts to reduce anxiety, and a sense of being stuck in the process. However, if these items were the end product to which the chosen items were thematically grouped, then I am missing a section of Results where you would explain how the chosen items were categorised into these three categories. If these themes were not the final “factors” in the list of the items, I am not sure how these categories were created.

Is the work clearly and accurately presented and does it cite the current literature?

Partly

If applicable, is the statistical analysis and its interpretation appropriate?

Not applicable

Are all the source data underlying the results available to ensure full reproducibility?

Yes

Is the study design appropriate and is the work technically sound?

Yes

Are the conclusions drawn adequately supported by the results?

Yes

Are sufficient details of methods and analysis provided to allow replication by others?

Partly

Reviewer Expertise:

Psychotherapy, randomized controlled trials, meta-analysis, common mental disorders

I confirm that I have read this submission and believe that I have an appropriate level of expertise to confirm that it is of an acceptable scientific standard, however I have significant reservations, as outlined above.

# Response

**Published:** 2026-06-26

**Authors:** Elizabeth Kirkham

**Affiliations:** School of Health in Social Science, University of Edinburgh, Edinburgh, UK

Thank you for your thoughtful comments – your input has made the paper more clear! Please find my responses to your specific comments below in italics. 

 Abstract:

 Major comments: It would be helpful to explain in one or two sentences what the background of research on excessive rationalization is and why it is important to create and study this concept, instead of focusing on, for example, optimization of existing treatments for OCD.

***Thank you, I have added this.***

 Plain Language Summary:

 Minor comments: “It is therefore important to understand more about OCD and how it works.” => Could you perhaps reformulate this sentence? I feel like “how it works” is a bit too general and informal for a peer-reviewed journal.

***I have updated this to “It is therefore important to understand more about OCD and the thought patterns that maintain it.”***

 Introduction:

 Major comments:

- Page 4, second last paragraph: You are referring to having held two meetings with paid community collaborators in an effort to develop the concept of excessive rationalisation, which eventually also coined this term. Were these meetings held by your research group, or by other researchers? If the latter, I would recommend adding a reference to this event (a study resulting from this event, or a personal communication). If it was held by your research group, I would say that it was already the first step of the current research, so it would be best of describe this in the Methods, and its Results in the Results, if it fits well. Alternatively, if there is already a study published, then you can use a reference to this study here.

***Apologies that this was unclear. The concept of excessive rationalisation initially came from the researcher’s knowledge of the literature and lived experience of OCD. The purpose of the stakeholder sessions was to assess whether the concept was relevant to other people with OCD, and to give more depth to the initial idea. These sessions were analogous to a discussion within a research group. As such data were not collected as the people involved were viewed as expert advisors rather than participants. Nevertheless, I agree that this process represents an early stage of the research project, and as such I have moved it to the Method section. ***

- Pages 4-5: I would also place the whole explanation of what Delphi study is and why to conduct it in the Methods. I would just jump in the Introduction from the background of the research question to the aims of the current study and make sure that all methods are described in the Methods.

***I have moved all description of the Delphi to the Methods.***

- Page 5: Similarly, I would completely remove the last paragraph of the Introduction and explain it in the Methods. The Introduction should be closed by a paragraph summarising the aims of the current study.

***Good point! I have moved the information from the last paragraph of the introduction into the Methods. ***

- I also think that the Introduction does not really clearly defined excessive rationalisation, so the reader gets a bit lost what the manuscript is actually about. Maybe you could place some of the explanations from Appendices 1 and 2 to the Introduction, because they illustrate very well what the manuscript focuses on.

*I **agree this would make the introduction clearer - I have drawn on the appendices to provide an overview within the Introduction.***

 Methods:

 Major comments:

- Screening: Were there other in/exclusion criteria for the participants, such as age?

***Yes, they were required to be over 18 and available to complete all four surveys during the study period. I have updated the manuscript to make this clear. ***

- Round 1: I feel like the pre-survey period is very extensively described, even though it is not really the focus of the current paper. Maybe you can only say that an inclusion criterion of the current study is that participants reported experiencing excessive rationalisation, and then move on to describe the participation of the study by the participants who met this criterion?

***Thank you for this comment. I can see that this is quite a verbose section, and have cut this section down. As the first reviewer felt that the screening phase was important to report I have kept the substance of the section. I hope that the updated version is an acceptable compromise between the two reviewers.***

 Results:

 Major comments:

- The paper states that 27 participants registered their interest, but then in the Results, it is written that 23 individuals participated. What happened with the 4?

***This has been clarified in the revised version (see Figure 1). Of the four, three did not provide data in Survey 1 and were therefore assumed to have dropped out of the project. The other participant completed Survey 1 but left all the qualitative questions blank. Given that the qualitative questions were a fundamental aspect of the research project, it was decided that this participant would not be invited to the follow-up surveys.***

 Discussion:

 Major comments:

 • ⁃ In the summary of the results, you are mentioning three themes that the included items can be categorised to: thought control, attempts to reduce anxiety, and a sense of being stuck in the process. However, if these items were the end product to which the chosen items were thematically grouped, then I am missing a section of Results where you would explain how the chosen items were categorised into these three categories. If these themes were not the final “factors” in the list of the items, I am not sure how these categories were created.

***This was intended to offer the researcher’s brief interpretation of the overall item pool. However, the way it was included in the Discussion implied that there were themes derived from the data. I have therefore removed this comment from the Discussion.***

# Reviewer report: Reviewer response for version 1

**DOI:** 10.21956/wellcomeopenres.28746.r155654

**Published:** 2026-05-19

**Authors:** Anirudh Bakam

**Affiliations:** 1 All India Institute of Medical Sciences - Bibinagar, Hyderabad, Telangana, India

**Peer Review: "Conceptualization of a novel cognitive process in obsessive-compulsive disorder: A Delphi study on excessive rationalisation"**

**Decision: Accept with Minor Revisions**

 This is a well-written, rigorously conducted and timely study addressing a genuinely underexplored dimension of OCD. Any clinician delivering CBT for OCD will immediately appreciate its relevance. The blurry boundary between excessive rationalisation, mental compulsions, and neutralisation is encountered routinely in clinical practice yet remains poorly operationalised, and the authors have addressed this thoughtfully in the Discussion. The difficulty of monitoring such internal mental processes objectively makes structured classificatory attempts of this kind especially valuable. The co-produced Delphi methodology is well-justified and the resulting 30-item pool is a meaningful output. The following minor points are offered for consideration.

1. A CONSORT-style flow diagram is strongly recommended (Schulz et al., 2010). It would efficiently resolve several questions at once, including how many individuals accessed the screening form, how many were screened out and why, how many registered interest, and attrition across all four rounds. This single addition would substantially improve methodological transparency.
2. The total number of individuals who accessed the screening form prior to the 27 who registered interest should be explicitly reported. This figure carries independent significance, as the proportion who recognised excessive rationalisation as relevant to their own experience is itself informative about the construct's prevalence within the OCD population.
3. Round 1 involved qualitative open-ended responses from which items were generated. The authors are encouraged to include an explicit statement regarding thematic saturation (Guest et al., 2006; Braun & Clarke, 2006), or reference established norms for Delphi panel sizes (Hasson et al., 2000), to reassure readers that 20 to 23 participants were sufficient to capture the full conceptual breadth of excessive rationalisation. In qualitative inquiry, saturation rather than sample size per se is the accepted benchmark for sufficiency, and an explicit statement to this effect would considerably strengthen the Methods section.
4. The treatment history breakdown is appreciated. It would be worth noting that participants with prior CBT exposure may already have been trained to recognise and modify rationalisation-type responses, potentially leading to underreporting or altered framing of excessive rationalisation compared to treatment-naive individuals. A brief acknowledgment of this would add nuance to the interpretation of the item pool.
5. The sample is predominantly female, White British, and UK-based. A brief acknowledgment of how this may limit the cultural and demographic generalisability of the item pool would be appropriate, particularly given the global burden of OCD highlighted in the Introduction.
6. The paper would benefit from closing with a more explicit and prominent statement that this work represents the first step in a broader programme of research, and that formal scale development including factor analysis, reliability testing, and clinical validation is intended to follow. This contextualises the current contribution appropriately and will help both clinicians and researchers understand the trajectory of this line of work.

**References**

 (refer 1,2,3,4)

Is the work clearly and accurately presented and does it cite the current literature?

Yes

If applicable, is the statistical analysis and its interpretation appropriate?

Not applicable

Are all the source data underlying the results available to ensure full reproducibility?

Partly

Is the study design appropriate and is the work technically sound?

Yes

Are the conclusions drawn adequately supported by the results?

Yes

Are sufficient details of methods and analysis provided to allow replication by others?

Partly

Reviewer Expertise:

My expertise in OCD, CBT, and qualitative research methodology enabled me to assess the key aspects of this manuscript comprehensively. Given the current qualitative and conceptual nature of the work, a statistician's review may not be essential at this stage, though one would be strongly recommended when the authors proceed to formal scale development and validation.

I confirm that I have read this submission and believe that I have an appropriate level of expertise to confirm that it is of an acceptable scientific standard.

## References

[1] Braun V, Clarke V. Using thematic analysis in psychology. Qualitative Research in Psychology. 2006;3(2):77–101.

[2] Guest G, Bunce A, Johnson L. How many interviews are enough? An experiment with data saturation and variability. Field Methods. 2006;18(1):59–82.

[3] Hasson F, Keeney S, McKenna H. Research guidelines for the Delphi survey technique. Journal of Advanced Nursing. 2000;32(4):1008–1015. 11095242

[4] Schulz KF, Altman DG, Moher D. CONSORT 2010 statement: updated guidelines for reporting parallel group randomised trials. BMJ. 2010;340:c332. 10.1136/bmj.c332PMC284494020332509

# Response

**Published:** 2026-06-26

**Authors:** Elizabeth Kirkham

**Affiliations:** School of Health in Social Science, University of Edinburgh, Edinburgh, UK

Thank you very much for your generous comments and clinical perspective. Please see below for the updates I have made in relation to your suggestions.

 1. This is a very helpful point, and I have added a diagram to this effect. As discussed further below, a large portion of the screening form responses appeared to be from bots, as they came in at exactly the same time (the pattern of responses from genuine participants was more spread out).

 2. This is a good point. It was difficult to decipher this because there were a large number of unfinished responses that came in at exactly the same time that were apparently bot responses (e.g. on 12/11/23 there were 15 responses at 12:14, 19 responses at 12:21, and 20 responses at 12:26). I have added a sentence to the paper to that effect. In future research I will endeavour to ask people with OCD whether or not they find excessive rationalisation to be relevant to their own experience. As the second reviewer felt that the screening phase should be less thoroughly described, I hope that this approach is a satisfactory compromise.

 3. Thank you for raising this – I did consider sample size when planning the project, but it was missing from the first version of the paper. I have now added information on appropriate Delphi sample sizes. A data-saturation focused approach would have been difficult to implement in this particular project due to the limited time available to recruit new participants between Survey 1 and Survey 2. Nevertheless, having checked the “Qualitative coding of Survey 1” file within the supplementary materials I am confident that appropriate data saturation was achieved.

 4. I have added this suggestion – in fact this is an area I would like to investigate further in the future.

 5. This is a limitation of the present work which has been added to the discussion.

 6. Thank you for this suggestion! I have added this to the end of the paper.
